# Orbital infection due to *Saksenaea loutrophoriformis* in an immunocompetent adolescent: A case report

**DOI:** 10.22034/cmm.2026.345464.1697

**Published:** 2026-06-26

**Authors:** Somayeh Dolatabadi, Mohammad Hasan Aelami, Mohammad Javad Najafzadeh, Mohammad Etezad Razavi, Mohammad Saeid Sasan, Jos Houbraken

**Affiliations:** 1 Department of Biology, Hakim Sabzevari University, Sabzevar, Iran; 2 Department of Pediatrics, Faculty of Medicine, Mashhad University of Medical Sciences, Mashhad, Iran; 3 Department of Parasitology and Mycology, Faculty of Medicine, Mashhad University of Medical Sciences, Mashhad, Iran; 4 Ophthalmology, Eye Research Center, Mashhad University of Medical Sciences, Mashhad, Iran; 5 Westerdijk Fungal Biodiversity Institute, Utrecht, The Netherlands

**Keywords:** Mucorales, DNA sequencing, *Saksenaea loutrophoriformis*, Case report

## Abstract

**Background and Purpose:**

Mucormycosis is a rare fungal infection which can cause acute, disfiguring, and angioinvasive infections in immunocompromised patients with high mortality and morbidity rates. However, this study aimed to present the case of a healthy 7-year-old female.

**Case presentation:**

An immunocompetent female with orbital mucormycosis caused by *Saksenaea loutrophoriformis* from Mashhad, Iran was admitted to the hospital. This rare infection was the consequence of an injury due to a tree branch. The causative agent was identified by histopathology, routine mycological methods, and DNA sequencing of the Internal Transcribed Spacer 1 and 2 regions, including the intervening 5.8S gene and the partial large subunit region D1 and D2.

**Results:**

The patient was successfully treated with surgical debridement and antifungal treatment of amphotericin B and oral posaconazole.

**Conclusion:**

Molecular diagnosis is crucial in the absence of sporulation to avoid misidentification and late diagnosis. This report of a rare infection in an immunocompetent host is valuable for awareness of clinicians.

## Introduction

Mucormycosis is a rare fungal infection caused by mucoralean fungi (order *Mucorales*, *Mucormycotina*), which can cause acute, disfiguring, and angioinvasive infections in immunocompromised patients [1]. Mucormycosis may present as sino-orbital, rhino-cerebral, pulmonary, cutaneous, gastrointestinal, or disseminated disease with high mortality and morbidity rates if not treated properly [1]. The underlying risk factors include uncontrolled diabetes mellitus, cancer, leukaemia, use of corticosteroids, antibiotics, renal insufficiency, trauma, burn wounds, desferrioxamine therapy, and Coronavirus disease [2-3].

Mucormycosis is caused by at least 35 different *Mucorales* species, mainly *Rhizopus*, *Mucor*, and *Lichtheimia*, accounting for up to 70-80% of all cases [4-5], whereas *Cunninghamella*, *Apophysomyces*, *Saksenaea*, *Rhizomucor*, *Cokeromyces*, *Actinomucor*, and *Syncephalastrum* are responsible for less than 1-5% of the reported cases [5].

*Saksenaea* was first isolated in 1953 from soil in India [6], and the first human infection due to *S. vasiformis* was described by Ajello et al. in 1976 [7]. Three species were classified in the genus *Saksenaea* by Alvarez et al. in 2010, namely, *S. oblongispora*, *S. erythrospora*, and *S. vasiformis*. However, revisions applying multilocus phylogeny (internal transcribed spacer [ITS], large subunit [LSU], and *tef-1α*) revealed that the genus Saksenaea was genetically heterogeneous and encompassed more species (namely, *S. erythrospora*, *S. loutrophoriformis*, *S. oblongispora*, *S. trapezispora*, and *S. vasiformis* complexes) [8].

In 2009, *Saksenaea loutrophoriformis* was introduced as a new species based on a strain isolated from the eye of a patient in Utah, USA(2009, D.A. Sutton, holotype CBS H-23041, cultures ex-type UTHSC 08-379=FMR 10674; ITS, LSU, and EF1-α sequences GenBank FR687330, HM776682, and HM776693, MycoBank MB820008) [8]. Moreover, another case was reported in a previous study, which was isolated from palate necrotic tissue in Chandigarh, India (2015, J. Chander, living cultures M-1012/15=FMR 14516; ITS, LSU, and EF1-α sequences GenBank LT796164, LT796165, and LT796166). Another strain was also reported in Iran, which was misidentified as *S. vasiformis* at the time [9]. Besides these few cases, no additional clinical cases of S. loutrophoriformis have been reported in the literature.

## Case presentation

A7-year-old Iranian female patient without any risk factors was admitted, presenting with proptosis with orbital inflammation in the left eye, pain, and fever. Incisional biopsy was performed, and histopathology test confirmed soft tissue lesions, chronic granulomatous inflammation with eosinophils, microabscess formation, and fungal infection. Her primary false diagnosis was aspergillosis.

A week later, she referred to Khatam Eye Hospital in Mashhad, Iran. Amphotericin B (150 mg/day) was prescribed; however, due to the cost and unavailability of this medicine during the COVID-19 pandemic and the rise in mucormycosis cases, voriconazole (250 mg/12 h) was administered as the second-line drug of choice.

Magnetic resonance imaging (MRI) showed an enhanced mass in the inferior aspect of the left orbit involving orbital soft tissue and extraocular muscles. Ophthalmic examination of the right eye was normal. Results of all blood tests were within normal limits, except for white blood cell count (WBC=14.2 9 10^3^/µl, elevated) and creatine (0.5 mg/dL, decreased). Treatment was continued based on aspergillosis. Her best-corrected visual acuities (BCVA) in both eyes were 10/10. Intraocular pressures were 28 mmHg in the right eye and 16 mmHg in the left eye.

Re-excision (Figure 1) was considered and showed non-septate hyphae indicative of mucormycosis. Biopsy showed fibro-connective tissue with extensive inflammation, multifocal necrosis, granuloma formation, and scattered broad aseptate fungal hyphae, while microscopy findings indicated mucormycosis in the orbit; therefore, voriconazole was discontinued.

**Figure 1:**
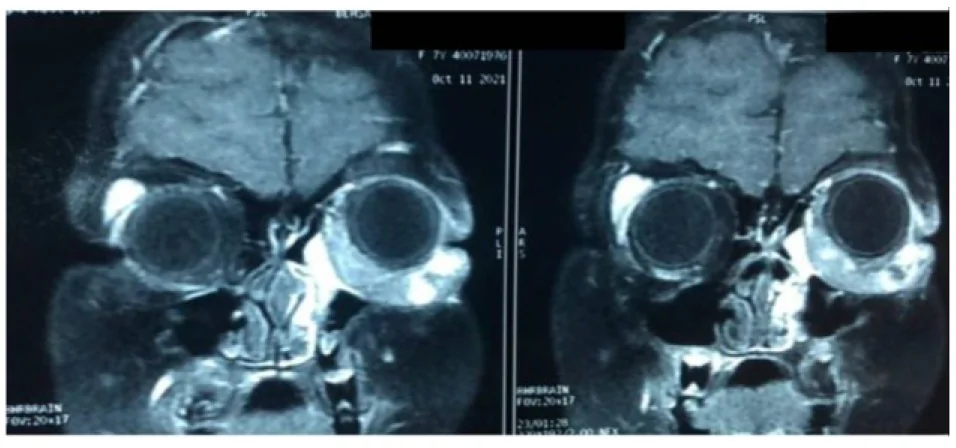
Magnetic resonance imaging showing enhanced mass in the inferior aspect of the left orbit involving orbital soft tissue and extraocular muscles.

The patient was hospitalized again with inflammation in the left eye, no fever or pain, with high WBC (14.12 103/µl) and low mean corpuscular volume (79.2).

Two weeks later, she was discharged with normal health and a normal orbital MRI. Debulking of the left orbit plus four months of amphotericin B (150 mg/day for 15 days) and 3.5 months of oral posaconazole (150 mg/day) were administered.

On follow-up after four months, BCVA of both eyes was 10/10. Ophthalmic examination and orbital MRI were normal. Biopsy specimens taken at the end of the treatment were negative for fungus (Figure 2).

**Figure 2:**
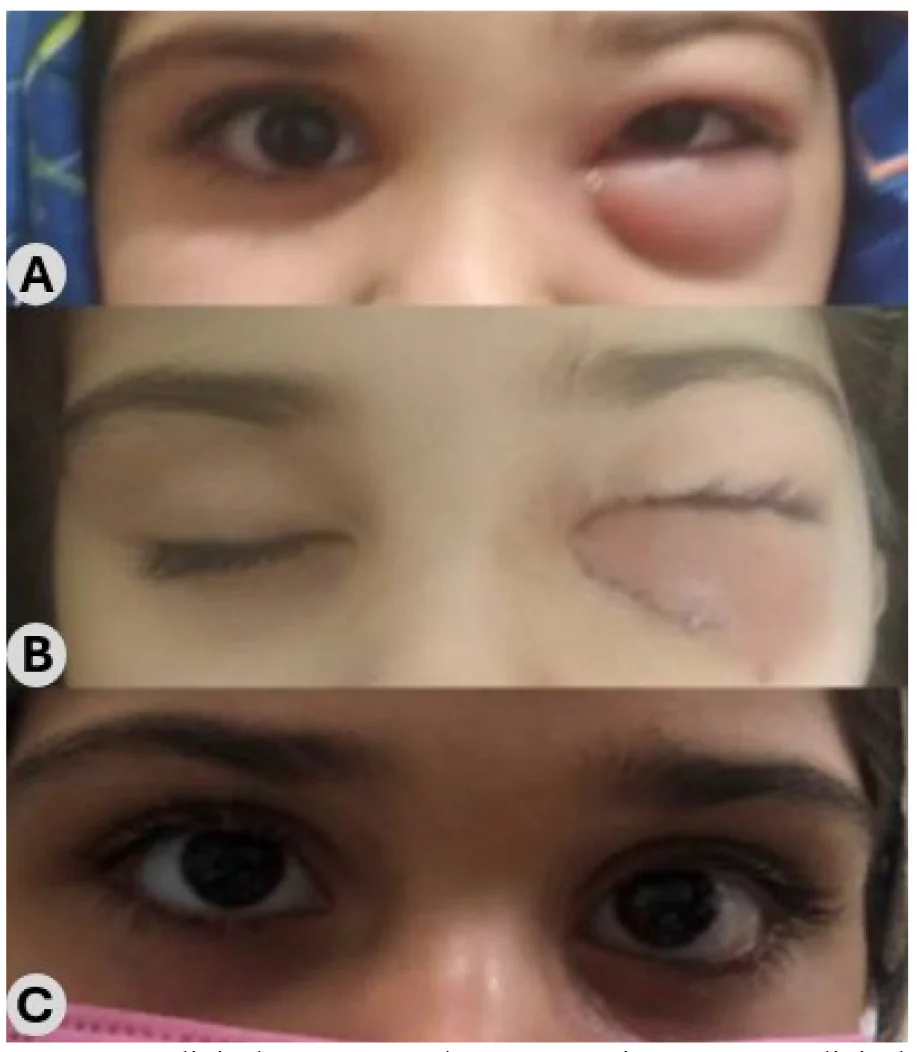
**A.** Clinical response at the pre-operative stage. **B.** Clinical response at the post-operative stage. **C.** Patient at the 4-month follow-up.

## Fungal morphological examination

The samplings were sent to microbiology and histopathology labs and were inoculated on Sabouraud’s dextrose agar (SDA, Merk, Germany) containing chloramphenicol (50 mg/L) and incubated at 30 ℃ and 37 ℃. The colony was observed after five days. Slide cultures revealed non-septate fungi, a typical flask-shaped sporangium with globular columellae arising from dichotomously branched darkly pigmented rhizoids at 400× magnification. No sporulation was recorded. For cytopathological examination, slides were stained with hematoxylin and eosin stain (H&E) and observed with 400× magnification.

## Fungal molecular identification

The strain was cultivated on malt extract agar (MEA, Merk, Germany) and DNA was extracted after a 3-day incubation period in the dark at 25 ˚C using the DNeasy UltraClean™ Microbial DNA Isolation Kit (QIAGEN, Germany). Fragments containing the ITS 1 and 2 regions and the 5.8S gene (ITS) and the partial large subunit region D1 and D2 (LSU) were amplified and sequenced. The used primers were LS266 and V9G for ITS, and LR0R and LR5 for LSU [10]. The polymerase chain reaction (PCR) fragments were sequenced in both directions with the primers used for PCR amplification using the ABI Prism® Big DyeTM Terminator v. 3.0 Ready Reaction Cycle Sequencing Kit. Samples were analyzed on an ABI PRISM 3700 Genetic Analyzer, and contigs were assembled using the forward and reverse sequences with the program SeqMan from the LaserGene package. The sequences were compared with those available in NCBI’s nucleotide database (GenBank) using BLAST and with the in-house sequence database of Westerdijk Fungal Biodiversity Institute. The ITS and LSU sequences were deposited in the GenBank database under accession numbers OQ550015 and OQ533697. The strain was deposited in the CBS collection housed at the Westerdijk Fungal Biodiversity Institute, Utrecht, the Netherlands, under number CBS 150004.

## Phylogenetic analysis

To study the phylogenetic relationship of the isolated Saksenaea isolate with other strains, the most related sequences were downloaded from GenBank using the BLAST algorithm (https://blast.ncbi.nlm.nih.gov/Blast.cgi). Sequences of identified species were downloaded with accurate nomenclature and environmental samples. All sequences were initially aligned, their alignments were manually adjusted, and ambiguous regions were excluded from the alignments using Mesquite (version 3.04). Phylogenetic relationships were determined by the Neighbor Joining algorithm using PAUP* (version 4.0). The substitutions/site rate was 0.005. Trees are depicted in Figure 3.

**Figure 3:**
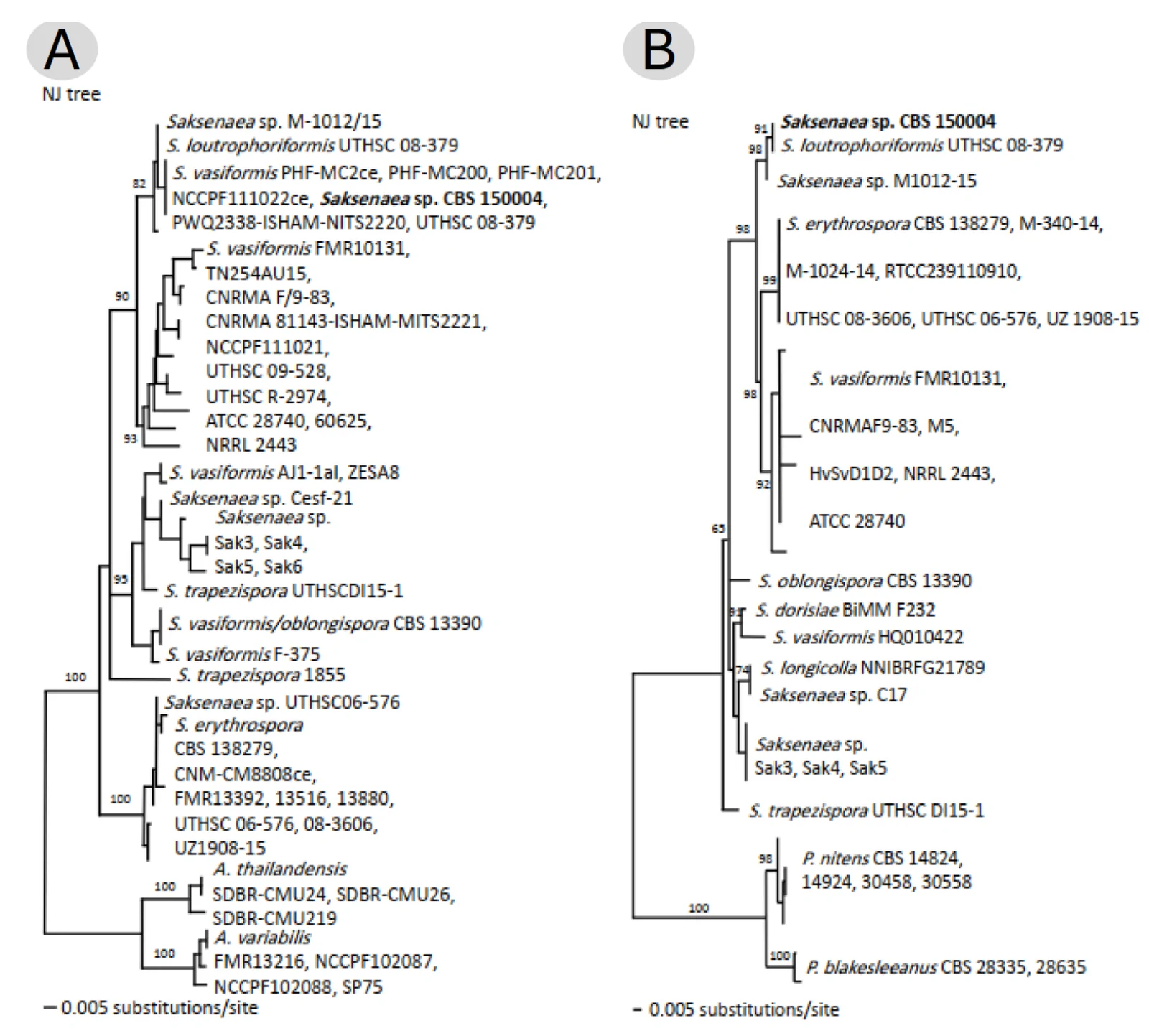
Neighbor joining trees based on (A) internal transcribed spacer region sequences and (B) the large subunit gene. Substitution/site=0.005.

## Discussion

An increasing incidence of fungal infections of the eye has been reported in specific geographic regions, due to certain fungal species [11, 12, 13]. Accurate and rapid diagnosis with a mycologist is essential for the treatment. *Saksenaea* is a rare soil fungus that mostly causes cutaneous and subcutaneous infections following trauma and a skin barrier break [7, 14]. The infection remains localized and, as such, responds well to the surgical debridement. However, rare cases of rhino-orbital, renal, or disseminated diseases have been reported [15].

Skin and soft tissue manifestations can include rapidly progressive cellulitis or necrotizing fasciitis, abscess formation, ulceration, or chronic lesions that progress over several months [16]. This infection has been reported in both immunocompromised and immunocompetent individuals and can vary from a mild chronic infection to a severe and fatal acute infection [14]. Samaras et al. 2019 [14] reported a total of 46 cases of *S. vasiformis*, the closest species to *S. loutrophoriformis*, including 30 male and 16 female patients. Most patients (n=35) were immunocompetent, and 29 of them recovered, while 17 died. In another study performed in 2022, a total of 65 cases were reported [16], 24 (37%) of whom died. In the aforementioned study, the most common clinical presentation was subcutaneous mucormycosis (60.9%) followed by rhino-orbito-cerebral mucormycosis (14%). Furthermore, cutaneous infection was reported after a scorpion sting in a 14-year-old immunocompetent adolescent [17] and also after a mild injury in a 39-year-old male [14].

Few cases of rhino-orbital infections have been reported, including a rare pediatric rhino-orbital infection caused by *S. vasiformis* [15], an orbital infection in an immunocompetent 22-year-old Thai female [18], and orbital cellulitis in an 11-year-old immunocompetent girl [19]. Reports of immunocompromised patients include a girl with acute lymphoblastic leukemia resulting in death [20], a case of otomycosis [21], and cutaneous infections in patients with diabetes and systemic lupus erythematosus [22].

*Saksenaea vasiformis* has a predisposition for tropical and subtropical climates, such as the United States [19], Qatar [15], Australia [20], and India [22]. Majority of *S. vasiformis* infections were reported from India and Australia in a review performed by Singh et al. [16]. Although *Saksenaea* is commonly found in soil worldwide, the number of published cases of infection is not proportionally high. This is probably explained by the fact that the fungus fails to sporulate in routine mycological culture media (e.g., Sabouraud dextrose agar, malt extract agar, and corn meal agar), causing a false relationship between the reported cases and the actual probable occurrence of *S. vasiformis* infections. In the present study, sporulation was not observed; therefore, the diagnosis may have been missed. When this infection is suspected, the fungi should be cultured on specific media to induce sporulation or identified by molecular tools. ITS sequencing has been reported as a reliable barcoding marker for *Mucorales*, and is to be considered as the standard for molecular identification of this fungal order [23].

Due to difficult identification and hence late diagnosis, a combination of surgery with antifungals, such as amphotericin B and/or posaconazole [19, 22], would be the common treatment [14-15]. Amphotericin B is the first-line drug, and lipid formulations are significantly less nephrotoxic and provide more safety at higher doses, while posaconazole, a new-generation triazole, can be used as an alternative to amphotericin B in the event of toxicity [19]. The present case was treated with debridement surgery in combination with amphotericin B and oral posaconazole [16] and showed a normal condition at the 4-month follow-up. Usage of posaconazole and surgical management was associated with significantly better survival [16]. This case has been the first well-documented case of *S. loutrophoriformis* from Iran.

## Conclusion

Mucormycosis mostly happens in immunocompromised patients with risk factors. However, in this case, an adolescent patient with no apparent health problem was affected following inoculation with a sharp branch. Reporting these kinds of cases will raise our awareness of the probability of these infections. Rapid diagnosis is crucial to increase the rate of morbidity and mortality, as well as hospital stay and consequent costs.
